# Diagnostic performance of wide-field optical coherence tomography angiography for high myopic glaucoma

**DOI:** 10.1038/s41598-023-49542-y

**Published:** 2024-01-03

**Authors:** Rim Kyung Hong, Ji Hong Kim, Gyungmin Toh, Kyeong Ik Na, Mincheol Seong, Won June Lee

**Affiliations:** 1 Department of Ophthalmology, Hanyang University College of Medicine, 222-1, Wangsimni-ro, Seongdong-gu, Seoul, 04763 Korea; 2 Department of Ophthalmology, Hanyang University Guri Hospital, Guri, Korea; 3 Department of Ophthalmology, Hanyang University Seoul Hospital, 222-1, Wangsimni-ro, Seongdong-gu, Seoul, 04763, Korea; 4 Department of Ophthalmology, Kangdong Sacred Heart Hospital, Hallym University College of Medicine, Seoul, Korea

**Keywords:** Diagnostic markers, Glaucoma

## Abstract

Diagnosing and monitoring glaucoma in high myopic (HM) eyes are becoming very important; however, it is challenging to diagnose this condition. This study aimed to evaluate the diagnostic ability of wide-field optical coherence tomography angiography (WF-OCTA) maps for the detection of glaucomatous damage in eyes with HM and to compare the diagnostic ability of WF-OCTA maps with that of conventional imaging approaches, including swept-source optical coherence tomography (SS-OCT) wide-field maps. In this retrospective observational study, a total 62 HM-healthy eyes and 140 HM eyes with open-angle glaucoma were included. Patients underwent a comprehensive ocular examination, including SS-OCT wide-field and 12 × 12 WF-OCTA scans. The WF-OCTA map represents the peripapillary and macular superficial vascular density maps. Glaucoma specialists determined the presence of glaucomatous damage in HM eyes by reading the WF-OCTA map and comparing its sensitivity and specificity with those of conventional SS-OCT images. The sensitivity and specificity of 12 × 12 WF-OCTA scans for HM-glaucoma diagnosis were 87.28% and 86.94%, respectively, while, the sensitivity and specificity of SS-OCT wide-field maps for HM-glaucoma diagnosis were 87.49% and 80.51%, respectively. The specificity of the WF-OCTA map was significantly higher than that of the SS-OCT wide-field map (*p* < 0.05). The sensitivity of the WF-OCTA map was comparable with that of the SS-OCT wide-field map (*p* = 0.078). The WF-OCTA map showed good diagnostic ability for discriminating HM-glaucomatous eyes from HM-healthy eyes. As a complementary method to an alternative imaging modality, WF-OCTA mapping can be a useful tool for the detection of HM glaucoma.

## Introduction

Glaucoma is characterized by progressive optic nerve damage resulting in irreversible visual field impairment^1,2^. Visual field impairment is preceded by increased optic nerve papilla depression and structural damage to the retinal nerve fiber layer (RNFL), perceptible symptoms do not arise until the disease has progressed to its final stages^3^. Glaucoma has poor prognosis in advanced stages and is difficult to treat with surgery and medication; thus, detecting structural damage in the optic disc and RNFL in the early stages of glaucoma is crucial for its diagnosis and treatment^4,5^.

Myopia is an ocular refractive error that is becoming more prevalent worldwide^6,7^. Myopia is a known risk factor for glaucoma, and several studies have reported an increased risk of ocular hypertension, normal-tension glaucoma, and primary open-angle glaucoma in patients with myopia. Therefore, diagnosing and monitoring glaucoma in patients with myopia are important^8–10^.

RNFL evaluation is helpful for the identification and follow up of glaucoma. Certain studies have investigated the diagnostic effectiveness of measuring RNFL thickness using optical coherence tomography (OCT)^11^. Clinically, OCT has been used to quantitatively identify structural glaucomatous damage and is helpful for early detection of glaucoma. However, in practice, a high false-positive rate is known for glaucoma diagnosis due to myopic eye changes, such as tilting, rotation, torsion, and peripapillary atrophy of the optic nerve head, in addition to the fact that identification of RNFL defects with conventional red-free photography is limited in myopia^12^.

In contrast to conventional fluorescein angiography, OCT angiography (OCTA) is a noninvasive, contrast-free method of viewing the microvasculature systems of the peripapillary and macular regions. It can measure blood flow and vessel density (VD) separately in the superficial capillary plexus (SCP) and deep capillary plexus^13^. Peripapillary retinal VD measured with OCTA was lower in glaucomatous eyes, and decreased VD in OCTA images can be associated with areas of RNFL defects^14,15^. OCTA is a widely known imaging tool that is valuable for sensing and quantifying glaucomatous damage and its progression, as OCTA, which combines peripapillary and macular areas, has been reported to help diagnose glaucoma in eyes with high myopia (HM)^16^. As technology advances make it commercially available for the visualization of a larger area in a single scan, OCTA is intended to be used for research on HM-glaucomatous eyes. Therefore, in this study, OCTA was performed on a 12 × 12 mm scan to obtain a superficial VD map of the peripapillary and macular regions. OCTA was also performed as a wide-field scan to analyze the peripapillary and macular regions, in contrast to that in previous studies.

The purpose of this study was to evaluate the diagnostic ability of the wide-field OCTA (WF-OCTA) and OCT wide-field maps for the detection of glaucomatous impairment in eyes with HM and to compare the diagnostic ability of the WF-OCTA map with that of the OCT wide-field map.

## Results

A retrospective study was conducted on 202 eyes of 101 patients. Sixty-two and 140 eyes were categorized as HM-healthy and HM-glaucomatous, respectively. The mean age of the patients was 54.35 ± 6.89 years in the HM-healthy eye group and 57.25 ± 7.73 years in the HM-glaucomatous eye group. The mean intraocular pressure (IOP) was 15.3 ± 3.01 mmHg in the HM-healthy eye group and 16.4 ± 2.49 in the HM-glaucomatous eye group, with an average axial length of 26.46 ± 1.11 mm and 26.67 ± 1.42 mm, respectively, and a spherical equivalent response of − 6.23 ± 2.01 D and − 6.79 ± 1.82 D, respectively. The percentages of previous cataract surgery were 19.02% in the HM-healthy eye group and 24.89% in the HM-glaucomatous eye group (Table 1). The percentages of previous refractive surgery were 12.90% and 7.14% in the HM-healthy eye group and HM-glaucomatous eye group, respectively (Table 1). No statistically significant differences were found between the two groups.

**Table 1:** Baseline characteristics of patients and parameter values of wide-field optical coherence tomography angiograpy (WF-OCTA) map and optical coherence tomography (OCT) wide-field map.

	HM-healthy eye (N = 62)	HM-glaucoma eye (N = 140)	*P* value
Sex (male:female, N)	32:30	66:74	0.284
Age (Years, range)	54.35 ± 6.89	57.25 ± 7.73	0.345
IOP (mmHg)	15.3 ± 3.01	16.4 ± 2.49	0.249
SE (Diopter)	− 6.23 ± 2.01	− 6.79 ± 1.82	0.412
Axial length (mm)	26.46 ± 1.11	26.67 ± 1.42	0.397
Cataract surgery (%)	19.02	24.89	0.484
Previous refractive surgery (%)	12.90	7.14	0.097
OCT wide-field map			
RNFL (peripapillary)			
RNFL total	98.11 ± 11.19	75.35 ± 16.33	< 0.001
RNFL superior	121.80 ± 23.07	97.57 ± 20.92	< 0.001
RNFL inferior	122.87 ± 16.51	85.05 ± 28.67	< 0.001
GCC			
GCC total	103.75 ± 7.09	90.18 ± 12.44	< 0.001
GCC superior	102.70 ± 6.96	95.89 ± 13.35	< 0.001
GCC inferior	103.75 ± 7.33	86.89 ± 14.76	< 0.001
GCIPL			
GCIPL total	67.28 ± 5.65	58.84 ± 8.95	< 0.001
GCIPL superior	67.78 ± 5.77	61.69 ± 10.66	0.003
GCIPL inferior	66.06 ± 4.83	55.21 ± 10.37	< 0.001
OCTA map			
VD of SCP (peripapillary)			
VD total	44.03 ± 2.48	41.57 ± 3.36	< 0.001
VD superior	44.15 ± 2.96	41.86 ± 3.95	< 0.001
VD nasal	41.58 ± 4.10	39.45 ± 4.59	< 0.001
VD inferior	43.73 ± 3.51	40.78 ± 4.14	< 0.001
VD temporal	46.65 ± 2.77	44.18 ± 4.89	<0.001

Values are presented as mean ± standard deviation (min–max).

*HM* high myopia, *IOP* intraocular pressure, *SE* spherical equivalent, *RNFL* retinal nerve fiber layer, *GCC* ganglion cell complex, *GCIPL* ganglion cell–inner plexiform layer.

The parameters of each test were divided into total, superior, and inferior areas for the RNFL, ganglion cell complex (GCC), and ganglion cell–inner plexiform layer (GCIPL) in the OCT wide-field map and subdivided into superior, nasal, inferior, and temporal areas of the VD in the peripapillary OCTA map, including the total average value. HM-glaucomatous and HM-healthy eyes were separately analyzed for both tests. All parameters, including the RNFL, GCC, GCIPL, and VD, showed significant differences between HM-glaucomatous eyes and HM-healthy eyes (Table 1).

**Figure 1:**
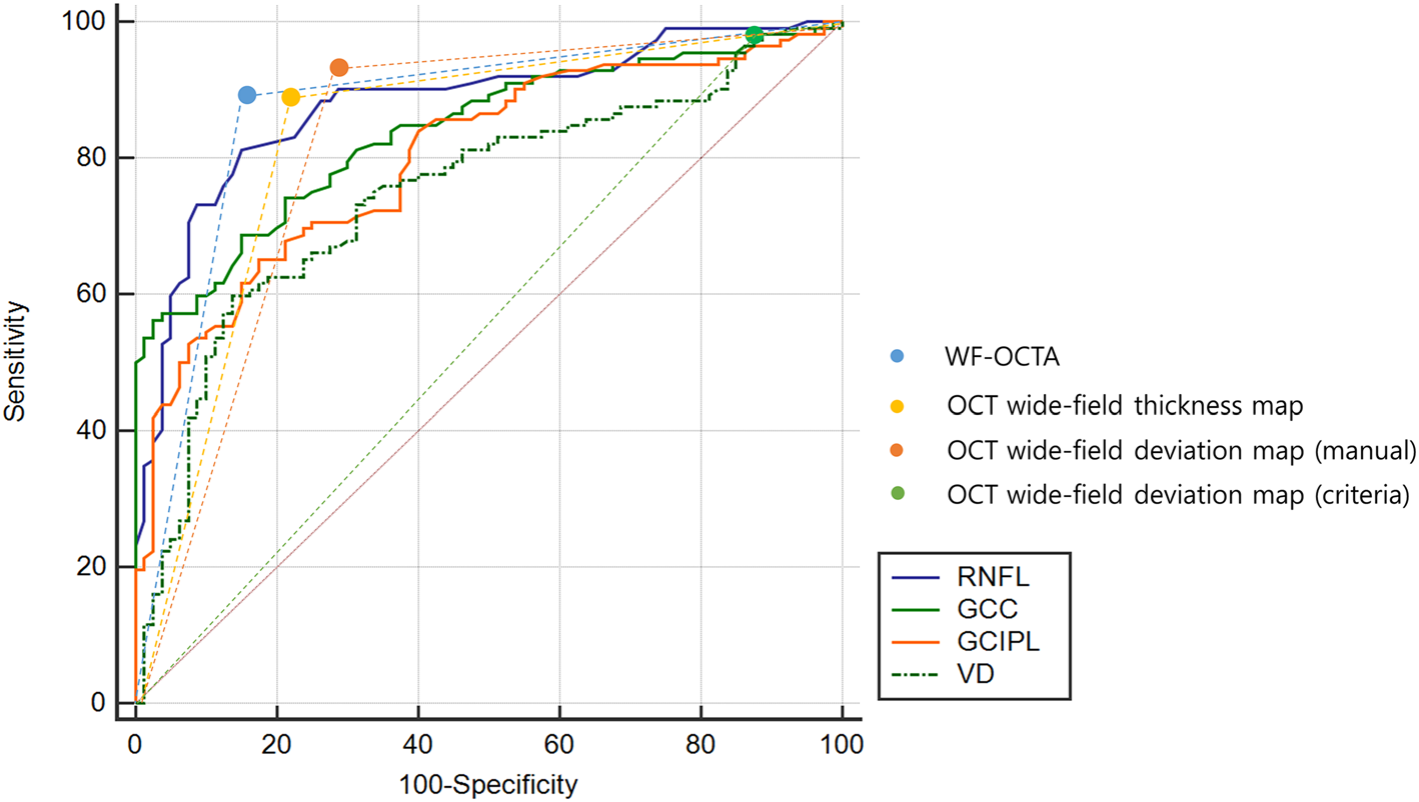
Comparison of the area under the receiver operating characteristic curves (AUROCs) for discriminating between glaucomatous eyes with high myopia (HM) and healthy eyes with HM. The AUROC values of retinal nerve fiber layer (RNFL, blue line), ganglion cell complex (GCC, green line), ganglion cell-inner plexiform layer (GCIPL, yellow line) thicknesses, and vessel density (VD, green dot line) were 0.880, 0.840, 0.800, and 0.750, respectively. The total RNFL thickness and mean VD value were used when calculating the AUROC.

The areas under the receiver operating characteristic curve (AUROCs) for each metric were 0.880, 0.840, 0.800, and 0.750 for the RNFL, GCC, GCIPL, and VD, respectively (Fig. 1). We used the total OCT thickness value and mean VD value when calculating the AUROC. The comparison of AUROC values is shown in the supplementary Table 1. The sensitivity and specificity of each imaging modality were compared and analyzed using McNemar’s test. The sensitivity of the WF-OCTA map for the diagnosis of glaucoma was 87.28 and specificity was 86.94. The OCT wide-field RNFL thickness map had a sensitivity of 87.49 and specificity of 80.51. The sensitivities of the OCT wide-field and WF-OCTA maps did not show a statistically significant difference; however, the specificity of the WF-OCTA map was significantly higher than that of the OCT wide-field map (*p* < 0.05) (Table 2). The specificity of the WF-OCTA map was also higher than those of other categorical variables including the OCT wide-field deviation map (manual and criteria) (*p* < 0.05) (Table 2).

**Table 2:** Comparison of sensitivity and specificity of WF-OCTA map and OCT wide-field map.

	Sensitivity (%)	*p* value*	Specificity (%)	*p* value*
WF-OCTA map	87.28		86.94	
OCT wide-field RNFL thickness map	87.49	0.078	80.51	** < 0.001**
OCT wide-field deviation map(manual)	94.28	0.579	78.17	** < 0.001**
OCT wide-field deviation map(criteria)	100.00	NA	10.2	** < 0.001**

*P value index compared with the WF-OCTA map based on McNemar’s test.

Bold values indicate statistical significance with *p* value less than 0.05.

*WF-OCTA map* wide-field optical coherence tomography angiography map, *OCT* optical coherence tomography.

**Figure 2:**
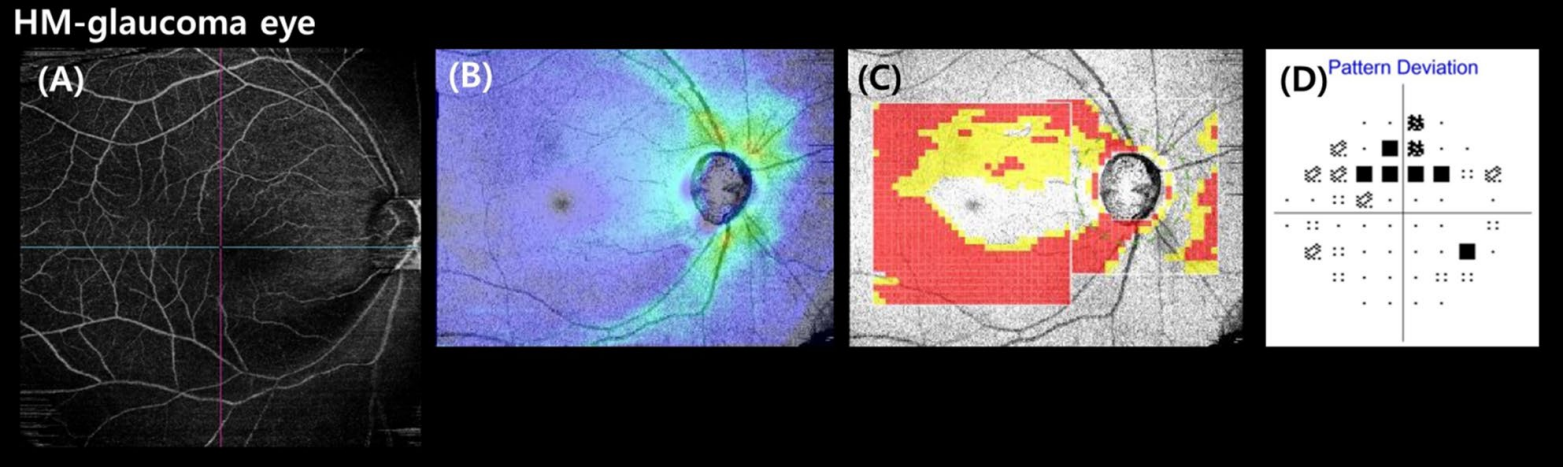
Case: Wide-field optical coherence tomography angiography (WF-OCTA) map showing an inferio-temporal retinal nerve fiber layer (RNFL) defect (**A**), consistent with that seen on the optical coherence tomography (OCT)-wide field RNFL thickness map (**B**) and OCT wide-field deviation map with a matching inferior-temporal RNFL defect (**C**). The WF-OCTA and OCT wide-field maps correspond to the impaired area in the visual field test (**D**). This case demonstrates the effectiveness of the WF-OCTA map in revealing an HM-glaucomatous defect.

**Figure 3:**
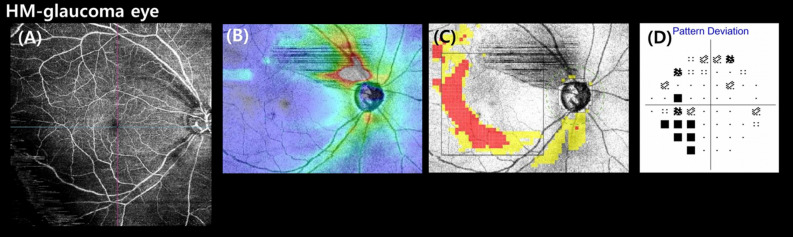
Case: In the case of HM-glaucomatous eyes with peripapillary retinoschisis, a definite margin of superior-temporal retinal nerve fiber layer (RNFL) thinning was visualized well in the wide-field optical coherence tomography angiography map (**A**) associated with an inferior-nasal visual field defect (**D**). The optical coherence tomography (OCT) wide-field RNFL thickness (**B**) and OCT wide-field deviation maps (**C**) for an HM-glaucomatous right eye with ambiguous superior-temporal RNFL thinning suggest false-negative results obtained with the OCT wide-field map.

A representative case showing the usefulness of the WF-OCTA map for detecting HM-glaucomatous defects is shown in Fig. 2. In addition, a case showing a HM-glaucomatous eye with peripapillary retinoschisis is shown in Fig. 3. The OCT wide-field map showed ambiguous RNFL thinning due to superotemporal peripapillary retinoschisis, but the WF-OCTA map showed a more definite RNFL defect area, which can be correlated with the matching area of the glaucomatous visual field defect in the Humphrey visual field test. This appeared to be a false-negative result in the OCT wide-field deviation map.

## Discussion

The present study compared the diagnostic accuracy of identifying glaucomatous defects in HM eyes between wide-field OCTA density (12 × 12 mm) and conventional wide-field maps derived from swept-source OCT (SS-OCT; 12 × 9 mm; Topcon DRI OCT). These data validated the clinical efficacy of wide-field OCTA maps for the diagnosis of HM glaucoma.

OCTA is a noninvasive imaging technique that can identify blood vessels in the retina. Earlier OCTA studies involving patients with glaucoma have shown that reduced peripapillary retinal perfusion can be observed as a focal defect, which can be quantified using the peripapillary flow index and VD, among others^17,18^. A previous study using OCTA for patients with glaucoma investigating longitudinal changes in RNFL defects, peripapillary and macular superficial retinal VD showed decreased superficial retinal microvasculature in patients with glaucomatous RNFL defects^19^. Another study on glaucoma used OCTA to assess the peripapillary circulation by observing microvasculature dropout in glaucomatous eyes, which is a sectoral capillary dropout in the peripapillary choroid^20^. In the macular and peripapillary areas, OCTA has been used to identify reduced superficial vascular complex, which is associated with visual field impairment in glaucoma^21^. A previous report attempted to reveal the diagnostic ability of the macular microvasculature assessed with SS-OCTA for HM-glaucomatous eyes and to compare it with that of macular thickness parameters assessed with SS-OCT using deep learning^22^.

Glaucomatous changes in eyes with HM are complicated to detect^23^. Moreover, identifying glaucoma using OCT remains a difficult task because distinguishing between glaucomatous structural impairment and measurement variability or myopia-related structural loss is difficult^20^. Hence, considerable effort has been expended to overcoming these problems when diagnosing HM glaucoma. Several studies have provided new methods to obtain better diagnostic ability for HM glaucoma with OCT by adjusting RNFL temporalization using the self-creating myopic normative database in the peripapillary or macular area^24–26^. Since the diagnosis of glaucoma in HM is challenging, several reports have introduced OCTA for HM-glaucoma diagnosis. Chang et al. reported that OCTA has a superior diagnostic ability to OCT for distinguishing HM eyes and HM-glaucomatous eyes by estimating peripapillary perfusion defect and macular perfusion^27^. Another study recommended the use of OCTA when diagnosing HM glaucoma; OCTA is better than OCT in detecting progression and evaluating risk of progression^28^. Efforts to integrate peripapillary and macula OCT images for detecting glaucoma have been made because both peripapillary and macular area imaging can be useful in glaucoma^29–33^. Based on this view, using OCT on a combination of peripapillary and macular areas, our team attempted to determine the advantages of the reconstructed WF-OCTA map by combining optic disc and macular OCTA density maps onto RNFL photography for distinguishing HM gluacoma^16^.

With technical advances, efforts have been made to visualize wider retinal areas, particularly particularly when the eye is afflicted with more than one retinal disease. These efforts have also been introduced to OCTA technology, and many reports have been conducted on WF-OCTA maps, such as studies demonstrating the practicability of wide-field en face SS-OCTA with extensive larger field imaging for the evaluation of the retinal vasculature in diabetic retinopathy^34^. Moreover, the WF-OCTA map has been used to investigate alterations in retinal microvasculature in eyes with preclinical diabetic retinopathy^35^. As previously mentioned, the WF-OCTA map is commonly used in the retinal area, but limited research is being conducted in the field of glaucoma.

In this study, (1) no statistically significant difference was found in the sensitivity between the OCT wide-field and WF-OCTA maps; however, the (2) specificity of the WF-OCTA map was significantly higher than that of the OCT wide-field map for the diagnosis of HM glaucoma. This result confirms that HM-glaucomatous eyes can be more specifically discriminated with the WF-OCTA map than with the conventional OCT wide-field map.

To the best of our knowledge, this study was the first to cover a wide area of the retina (12 × 12 mm) using OCTA for glaucoma. The WF-OCTA map combines the advantages of wide-field imaging and OCTA. Similar to OCT with the existing wide-field imaging, using OCTA to visualize a large area in a single scan could be valuable for analysis, whereas OCT could result in many false-positive findings, such as in HM. It has been reported that it is more beneficial in diagnosing glaucoma than conventional OCT even when using OCT wide-field map^30,31,33^: (1) detection of glaucomatous damage earlier or in greater detail, (2) intuitive display with single scan so images can be captured in an at-a-glance print-out, (3) more sensitive visualization of the temporal margin of RNFL defects and RNFL defects that are separate from the optic disc, and (4) enhanced visualization of RNFL defects that are not well covered by conventional small-field imaging (inferoinferior or superotemporal defects)^36–40^.

If we manage wider OCTA, reduction in superficial VD due to RNFL defects is more pronounced than when separately analyzing the optic nerve and macula, and the structural continuity of the two areas is suitable for intuitively identifying RNFL defects.

In the case of the OCT wide-field deviation map, it cannot reflect the thinner retina of an elongated eye due to myopia, compared with the map derived from non-HM healthy eyes. This results in numerous false positives. This is one of the reasons why HM was called “red disease” in previous studies^41^. The low specificity of OCT in HM-glaucoma diagnosis could be overcome by adopting the WF-OCTA map (Supplementary Fig. 1). Moreover, reducing the false-negative outcomes of OCT in detecting HM glaucoma is an additional benefit of OCTA technology. As shown in Fig. 3, which shows a case of retinoschisis, the OCT wide-field map revealed no abnormal signal in either the thickness or deviation maps; however, the WF-OCTA map showed a clear RNFL defect. Similar to the case of peripapillary retinoschisis, another benefit can be observed with OCTA, which is hard to be obtained by sole OCT imaging.

To realize the aforementioned advantages, a previous study was conducted to help diagnose glaucoma in HM while manually combining the area of the optic disc with macular OCTA^16^. The wide-field OCTA imaging used in this study had several advantages over wide-field OCTA imaging applications reported in previous studies: a wider range was obtained with a single shot and recombination, which was acquired separately, was not required. Additionally, we obtained a broader area than the previously obtained images of the optic nerve or macula. While being able to obtain information in a wider area than the present 12 × 9 mm imaging, the structural functional correlation such as in the “nasal step” area may be more relevant than that of the preceding studies for detecting glaucoma in HM, which will be evaluated further.

Despite the aforementioned advantages, the WF-OCTA map has limitations. Obtaining high-quality images from patients with mydriasis is time consuming. Moreover, more time is required to cover a wider area than with the currently used OCTA protocol. This leads to more blinking and complicates focusing; thus, image acquisition that is effortless for both patients and examiners is challenging in clinical practice.

The present study has the following limitations. First, we only determined whether the peripapillary and macular capillary densities decreased. For this reason, quantitative analysis should be conducted in the future because only the presence or absence of peripapillary and/or macular capillary density reduction was evaluated. In commercially available built-in software, the 12 × 12 OCTA scan report overlapped the RNFL or GCIPL/GCC thickness map of the 12 × 12 area on the OCTA image (Supplementary Fig. 2). If a WF-OCTA density map with color-coded vascularity or a normative database of wide-field OCTA will be commercialized in the future, it may become a more useful imaging tool for the diagnosis and follow up of HM-glaucomatous eyes. Further analysis of the sequential relationship between the WF-OCTA and OCT wide-field maps is expected to facilitate the understanding of the pathophysiology of myopia and glaucoma. Direct comparison of the diagnostic performance between parameters (RNFL/GCIPL/GCC/VD value) and the wide-field OCTA map is difficult with existing statistical methods. We briefly tried to compare the diagnostic performance indirectly and emphasize the usefulness of wide-field OCTA “map as an image.”

In conclusion, the WF-OCTA map is a new imaging method that shows comparable diagnostic power to that of already employed OCT protocols and, ultimately, has the advantage of reducing false-positive findings in identifying glaucoma in patients with HM, which is challenging for clinicians. If quantitative analysis is supplemented with software development, it is expected to be more valuable in the future.

## Methods

This study was approved by the Institutional Review Board of Hanyang University Seoul Hospital and complied with the Declaration of Helsinki (IRB approval number: 2021-07-036-001). The requirement for informed consent was waived by the institutional review board of our institution owing to the study’s retrospective nature. The study design adhered to the tenets of the Declaration of Helsinki for biomedical research.

### Participants

We retrospectively reviewed the medical records of patients with HM and open-angle glaucoma and those with normal-tension glaucoma, including HM-healthy patients, who visited the glaucoma clinic of the Department of Ophthalmology, Hanyang University Hospital, from April 2022 to December 2022.

Patients with HM with an axial length of ≥ 26 mm or a spherical equivalent of ≤ − 6.5 diopters were included in this study. Glaucoma was defined as the presence of characteristic changes in the optic disc (neuroretinal rim notching or thinning, increased cupping, or a cup-to-disc ratio difference > 0.2 between the right and left eyes) and RNFL defects on red-free RNFL photography, which corresponded to glaucomatous visual field defects^16,30^. RNFL defects were characterized as diverging, arched, or wedge-shaped, and wider than the major retinal vessel at a distance of 1 disc diameter from the disc's edge^16,30^.

We divided the patients into groups of HM-healthy eyes without accompanying eye diseases and HM-glaucomatous eyes with open angles regardless of IOP. Only perimetric glaucoma was included to exclude ambiguous cases; preperimetric glaucoma, which is defined as the presence of characteristic glaucomatous changes in the optic disc and RNFL without visual field defects, was excluded^42^.

All patients underwent best-corrected visual acuity testing, slit-lamp examination, and IOP measurements using Goldmann applanation tonometry, SS-OCT, and OCTA (DRI OCT Triton; Topcon, Tokyo, Japan). Patients with a history of ocular surgery, other than for cataract, or of refractive surgery, including for ocular trauma and other chronic ocular conditions that could affect vision, and who had undergone retinal laser treatment or had a neurological condition that could result in changes in vision or the visual field, were excluded from the study. Patients were also excluded if their images had severe motion artifacts due to fixation loss or if their SS-OCT image quality was < 45.

The axial length was measured with IOL Master biometry (Carl Zeiss Meditec Inc., Dublin, CA, USA) and an automated visual field test using standard automated visual field analysis (Humphrey Visual Field Analyzer, SAP, 24-2 Swedish Interactive Threshold Algorithm Standard strategy; Carl Zeiss Meditec Inc., Dublin, CA, USA). We used two consecutive reliable visual field examinations. The visual field test was reliable with a visual field fixation loss rate of ≤ 20% and a false-positive and -negative rate of ≤ 15%. Glaucomatous visual field defects were defined as a threshold of three or more points on the pattern deviation plot at < 5% of normal on two consecutive examinations, with one point being less than 1%, or an outside normal limit on the glaucoma hemifield test, or a pattern standard deviation of < 5%. Normal visual field test results were defined as those with mean deviation and pattern standard deviation within 95% confidence interval and as normal according to the glaucoma hemifield test.

### Swept‑source OCT wide-field map

We used a wide-field scan protocol (12 × 9 mm) with a deep range imaging (DRI)-OCT device. DRI-OCT is an SS-OCT device that can visualize the peripapillary and macular areas. The peripapillary RNFL, macular GCC, and macular GCIPL were estimated. The thickness of each layer was measured by dividing them into total, superior, and inferior sections. We procured the OCT wide-field thickness map and OCT wide-field deviation map, as described in detail in a previous study^16,30^.

### WF-OCTA imaging

OCTA was performed on a scan of 12 × 12 mm (wide-field OCTA map, WF-OCTA map) with automatically segmented layers, generating en face images of the retinal vasculature. A superficial slab was selected from several segmented slabs, and an image of the superficial retinal segment of the corresponding area was obtained. SS-OCTA has a 20 µm lateral resolution. The volumes were obtained covering a 12 × 12 mm field-of-view, with the fovea centered. The scan speed was 100,000 A-scans per second. The VD around the optic nerve was obtained by considering an area of 4.5 × 4.5 mm. We evaluated the VD of the SCP divided into four groups and analyzed them as superior, nasal, inferior, and temporal to assess for decreases in the VD of the SCP.

### Definition of the RNFL defect

RNFL defects on the OCT wide-field map, including the OCT wide-field RNFL thickness map and OCT wide-field deviation map, have been explained comprehensively in a preceding report^30^. In the OCT wide-field RNFL thickness map, the RNFL defect is defined as an arched or wedge-shaped diverging dark blue area encompassing a gradual color scale alteration that seems to be less thick than adjacent regions on color-coded maps^16^. An RNFL defect on the SuperPixel map is defined as the presence of a wedge-shaped region of at least 20 contiguous yellow/red pixels matched with RNFL thinning in the OCT wide-field deviation map^16,30^. Many yellow and red pixels exist in the OCT wide-field deviation map in eyes with HM. Therefore, the evaluation of whether the red and yellow pixel pattern is meaningful was performed by two glaucoma specialists (manually) and according to the criterion of 20 contiguous pixels to improve accuracy^16^. RNFL defects on the WF-OCTA map were also defined as arcuate or wedge-shaped dark areas. The defect areas of each WF-OCTA and OCT wide-field map were correlated with the visual field defects to determine the presence or absence of glaucoma. The images were analyzed by two glaucoma specialists (W.J.L. and K.I.N.), and the senior’s (M.S.) judgment was considered when opinions differed.

### Diagnostic ability of OCT wide-field map and WF-OCTA: sensitivity and specificity

The specificity and sensitivity of the OCT wide-field and WF-OCTA map were analyzed for each test. The categorical OCT variables included OCT wide-field RNFL thickness maps and wide-field deviation maps (according to manual ratings and criteria). AUROC for each indicator was calculated and compared statistically to determine the diagnostic powers of the RNFL, GCC, GCIPL, and VD.

### Statistical analysis

Statistical analysis was performed using SPSS (version 26.0; IBM Corp., Armonk, NY, USA) and MedCalc (MedCalc Software Inc., Mariakerke, Belgium), and a *p* value < 0.05 was considered statistically significant. We used MedCalc software and the Delong test to compare the AUROC values^43^. The measurements of each image were compared using the chi-square and independent t-tests. The McNemar test was performed to compare the sensitivity and specificity of the WF-OCTA map, OCT wide-field thickness map, and OCT wide-field deviation map.

### Meeting presentation

This study was presented as a poster at the 2023 World Glaucoma Congress (WGC) in Rome.

### Supplementary Information

Supplementary Figure 1.

Supplementary Figure 2.

Supplementary Table 1.

## Data Availability

The datasets used and/or analyzed during the current study are available from the corresponding author on reasonable request.
