# Supplementary material for: Diagnostic performance of wide-field optical coherence tomography angiography for high myopic glaucoma
Source: Sci Rep. 2024 Jan 3;14:367. doi: 10.1038/s41598-023-49542-y (PMC10764299; doi:10.1038/s41598-023-49542-y)

Supplementary Figure 1. Case: HM-healthy eye with no retinal nerve fiber layer (RNFL) thinning in the wide-field optical coherence tomography angiography map (A) related to normal visual field test (D). The optical coherence tomography (OCT) wide-field RNFL thickness map (B) shows nonspecific findings, but the OCT wide-field deviation map (C) shows contiguous yellow/red pixel, which is a false-positive result.

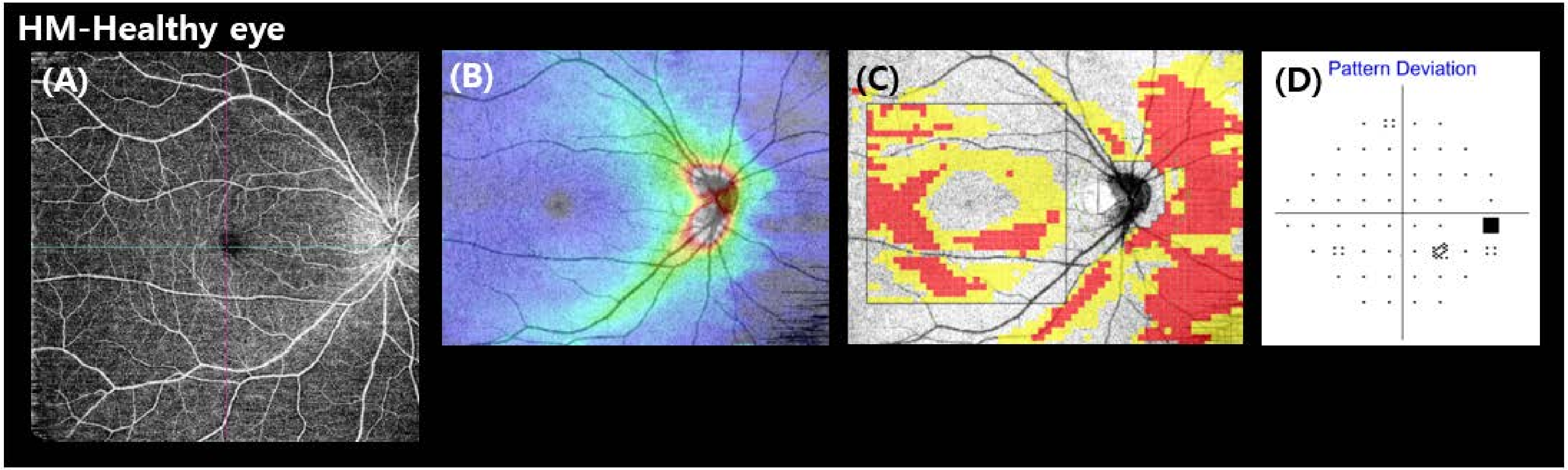

Supplement: Supplementary file 1 — Supplementary Figure 1. [file 41598_2023_49542_MOESM1_ESM.pdf]
