# Supplementary material for: Diagnostic performance of wide-field optical coherence tomography angiography for high myopic glaucoma
Source: Sci Rep. 2024 Jan 3;14:367. doi: 10.1038/s41598-023-49542-y (PMC10764299; doi:10.1038/s41598-023-49542-y)

Supplementary Figure 2. In commercially available built-in software, the wide-field optical coherence tomography angiography (WF-OCTA) scan report overlaps the retinal nerve fiber layer (RNFL) (A) and ganglion cell complex (GCC) thickness (B) map of the 12×12 area on the OCTA image (C).

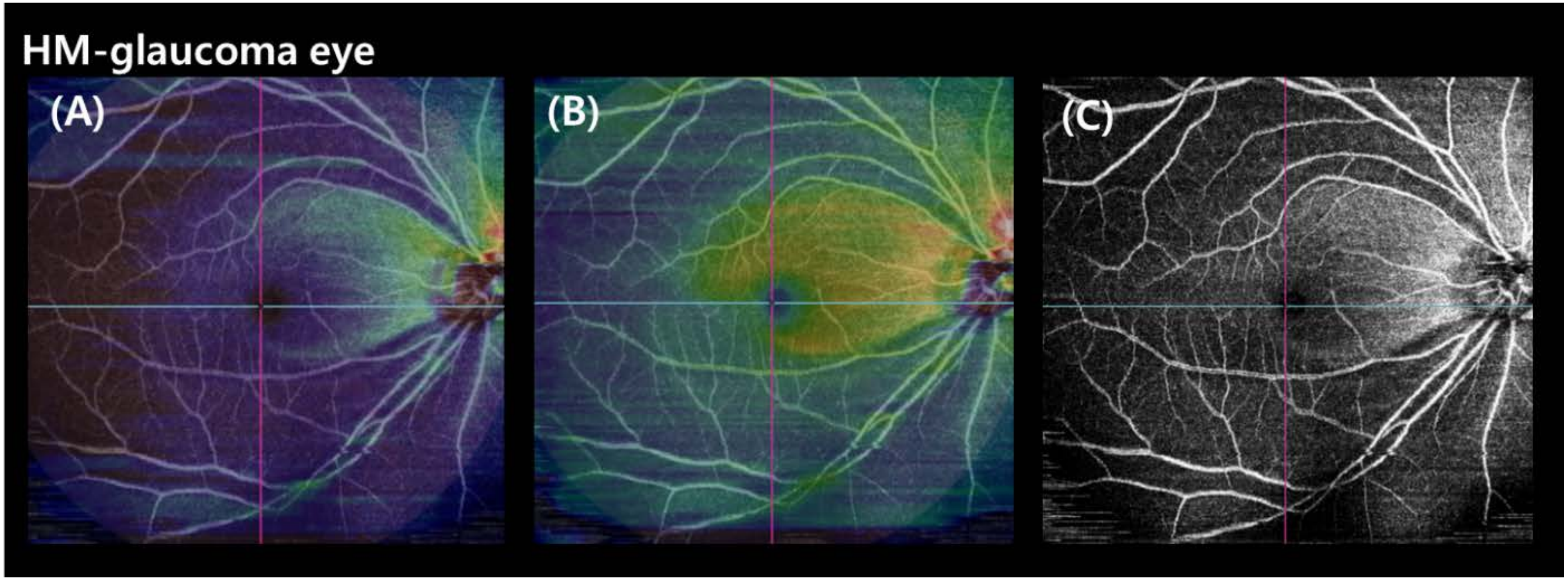

Supplement: Supplementary file 2 — Supplementary Figure 2. [file 41598_2023_49542_MOESM2_ESM.pdf]
