# Supplementary material for: Diagnostic performance of wide-field optical coherence tomography angiography for high myopic glaucoma
Source: Sci Rep. 2024 Jan 3;14:367. doi: 10.1038/s41598-023-49542-y (PMC10764299; doi:10.1038/s41598-023-49542-y)
Supplement: Supplementary file 3 — Supplementary Table 1. [file 41598_2023_49542_MOESM3_ESM.pdf]

Supplementary Table 1. Testing difference in AUC between the OCT parameters

	RNFL	GCC	GCIPL	VD
AUC (95% CI)	0.880 (0.825-0.922)	0.840 (0.780-0.889)	0.800 (0.737-0.854)	0.750 (0.682-0.809)
RNFL	NA	0.0398 P=0.1454	**0.0797 P=0.0019**	**0.130 P<0.0001**
GCC	0.0398 P=0.1454	NA	**0.0400 P=0.0298**	**0.0903 P=0.0238**
GCIPL	**0.0797 P=0.0019**	**0.0400 P=0.0298**	NA	0.0504 P=0.1639
VD	**0.130 P<0.0001**	**0.0903 P=0.0238**	0.0504 P=0.1639	NA

RNFL = retinal nerve fiber layer, GCC = ganglion cell complex; GCIPL = ganglion cell–inner plexiform layer; VD = vessel density; AUC= area under the curve; CI – confidence interval; NA = not available

Difference between areas and P values are shown. The method described by DeLong et al was used for AUC comparisons.
